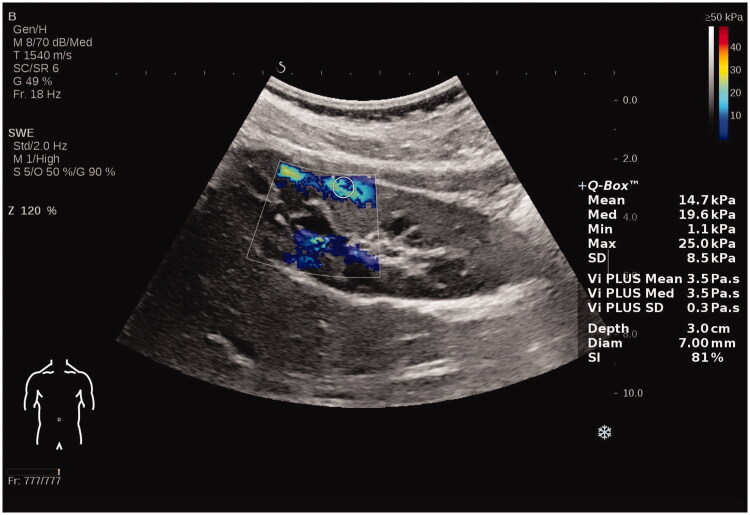# Letter to the editor regarding the article of Chen et al.

**DOI:** 10.1080/0886022X.2022.2077762

**Published:** 2022-05-23

**Authors:** Mehmet Sami Islamoglu, Sibel Gulcicek, Nurhan Seyahi

**Affiliations:** a Department of Internal Medicine, Cerrahpasa Medical Faculty, Istanbul University, Istanbul, Turkey; b Nephrology, Istanbul Training and Research Hospital, Istanbul, Turkey; c Division of Nephrology, Department of Internal Medicine, Cerrahpasa Medical Faculty, Istanbul University, Istanbul, Turkey

Dear Editor,

We read the letter of Chen et al. with interest and we thank the authors for pointing out an inadvertent error in our manuscript [1]. We noticed that we have sent the wrong figures which belong to our another study on elastography. Therefore, we are sending you the figures that we have taken with the Aixplorer (SuperSonic Imagin, Aix-en-Provence, France) brand device which we used in our study [2]. No changes are needed in the figure legends. Chen et al. made comments about the methodology of our manuscript and pointed out different results from the literature. Elastography is a new tool and standardization of methodology will add to its clinical applications. Our study and this letter at least raised attention to this issue. We think that constructive criticism raised by the authors will be a guide for further studies on this subject.

**Figure:**
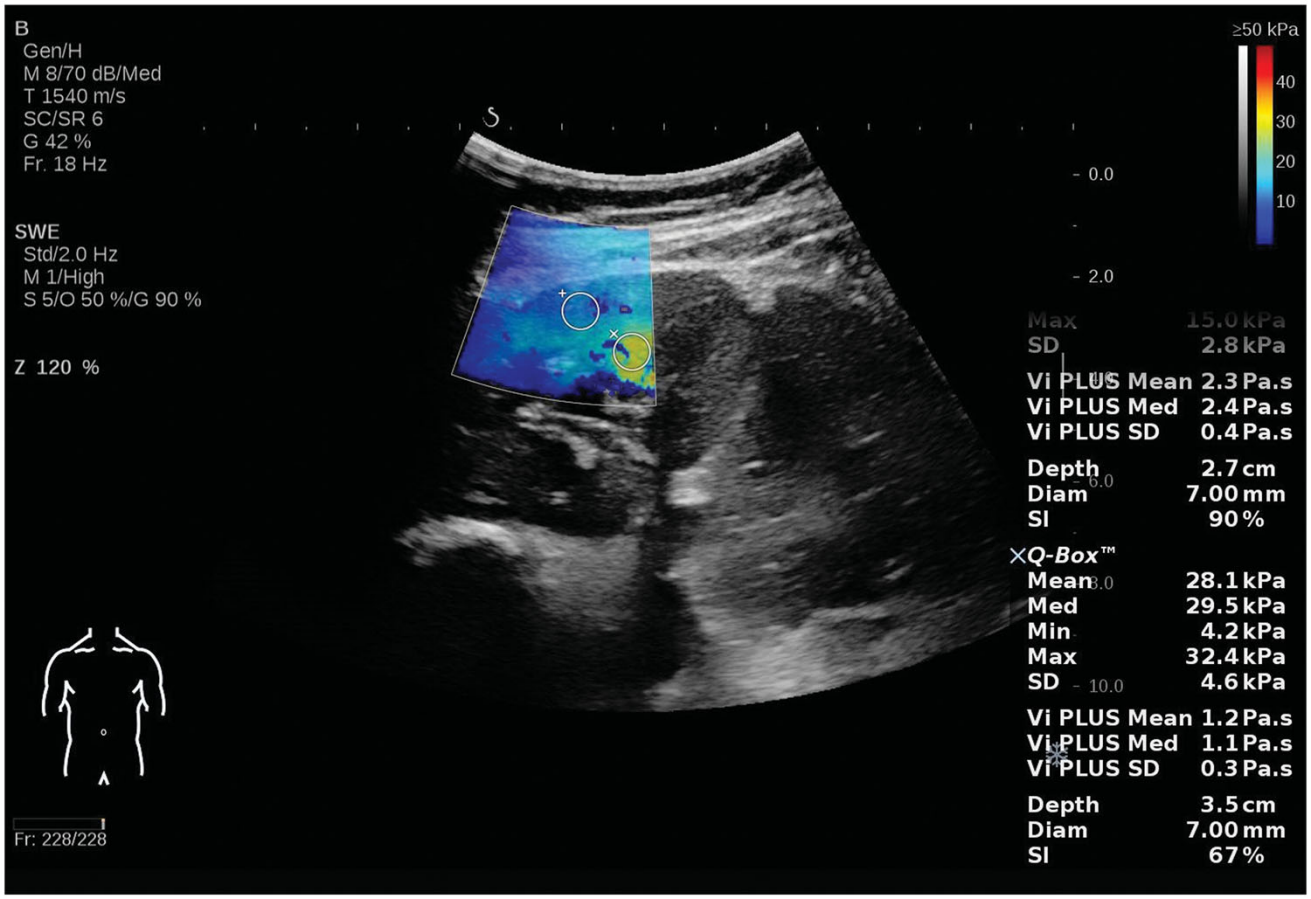

**Figure:**